# Transposable elements employ distinct integration strategies with respect to transcriptional landscapes in eukaryotic genomes

**DOI:** 10.1093/nar/gkaa370

**Published:** 2020-05-22

**Authors:** Xinyan Zhang, Meixia Zhao, Donald R McCarty, Damon Lisch

**Affiliations:** Shenzhen Branch, Guangdong Laboratory for Lingnan Modern Agriculture, Genome Analysis Laboratory of the Ministry of Agriculture, Agricultural Genomics Institute at Shenzhen, Chinese Academy of Agricultural Sciences, Shenzhen 518120, China; Department of Botany and Plant Pathology, Purdue University, West Lafayette, IN 47907, USA; Department of Biology, Miami University, Oxford, OH 45056, USA; Horticultural Sciences Department, University of Florida, Gainesville, FL 32611, USA; Department of Botany and Plant Pathology, Purdue University, West Lafayette, IN 47907, USA

## Abstract

Transposable elements (TEs) are ubiquitous DNA segments capable of moving from one site to another within host genomes. The extant distributions of TEs in eukaryotic genomes have been shaped by both *bona fide* TE integration preferences in eukaryotic genomes and by selection following integration. Here, we compare TE target site distribution in host genomes using multiple *de novo* transposon insertion datasets in both plants and animals and compare them in the context of genome-wide transcriptional landscapes. We showcase two distinct types of transcription-associated TE targeting strategies that suggest a process of convergent evolution among eukaryotic TE families. The integration of two precision-targeting elements are specifically associated with initiation of RNA Polymerase II transcription of highly expressed genes, suggesting the existence of novel mechanisms of precision TE targeting in addition to passive targeting of open chromatin. We also highlight two features that can facilitate TE survival and rapid proliferation: tissue-specific transposition and minimization of negative impacts on nearby gene function due to precision targeting.

## INTRODUCTION

Transposable elements (TEs) are ubiquitous mobile DNA sequences that can make additional copies of themselves and integrate into new positions in host genomes. Because of these properties, TEs can make up the majority, sometimes the vast majority, of eukaryotic genomes (1). Indeed, the overall architecture of many genomes is determined in large measure by the quantity and distribution of TEs, which in turn is determined by both integration preferences and selection following integration.

According to their structural and biochemical features, TEs can be classified as retrotransposons (Class I TEs) or DNA transposons (Class II TEs). Both Class I and Class II TEs can be either autonomous or non-autonomous. Autonomous elements encode proteins that are capable of mobilizing both autonomous and non-autonomous elements. Non-autonomous elements can only transpose in the presence of their cognate autonomous elements. Retrotransposons duplicate via a ‘copy-and-paste’ mechanism involving reverse transcription as a step in the process of replication. DNA transposons transpose via a ‘cut-and-paste’ mechanism, in which an element is physically excised from one position and reintegrated at a second position.

TEs have a significant impact on genome evolution. Increases in TE copy numbers result in increased genome size, complexity and instability (2). TE transposition is associated with chromosomal structural variation (3) and can also affect expression of individual genes (4). TEs have served as a rich source of novel mutations on which selection can operate and have contributed to gene evolution and phenotypic diversification (5). Despite this, changes induced by TEs are primarily neutral or deleterious to their hosts (6), and TEs are kept under strict control by host immune systems. Overlapping transcriptional and post-transcriptional silencing mechanisms have evolved in plants and animals as layered defenses that have evolved to repress TE expression and amplification (7,8). Although this system is highly efficient and results in epigenetic silencing of most TEs in most genomes, it is clear that TEs can also undergo rapid increases in copy number, and currently or recently active TEs have been identified in a wide variety of organisms (9).

The consequences of TE activity depend largely on where TEs integrate. While TE integration sites in eukaryotic genomes are broadly distributed, different TEs adopt distinct integration strategies, resulting in dramatically different insertion profiles (10). There is ample evidence that both DNA transposons and retrotransposons insert non-randomly in host genomes. For instance, in maize, *Activator* elements preferentially target linked genic regions (11), and maize *Mutator* (*Mu*) elements target unlinked open chromatin regions near recombination hot spots, which tend to be in the 5′ ends of genes (12). *P* elements in *Drosophila* integration has been associated with replication origins, which are also in the 5′ end of genes (13). Integration of L1 retrotransposons in the human genome also appears to be influenced by DNA replication, and is not targeted to either actively transcribed regions or to open chromatin (14,15). Some other retrotransposons, such as *Ty1* in yeast, target nucleosome-bound DNA near the H2A/H2B interface upstream of Pol III-transcribed genes due to physical interaction between the transposase and RNA polymerase III subunits (16–19). In contrast, gene space is a preferred target of many TEs (10), presumably because these are regions of relatively open chromatin, which may facilitate both integration and subsequent expression of autonomous elements (20,21). However, this is not always the case. *Tal1*, for instance, targets centromeres (22) and *Ty5* targets silenced heterochromatin (23). Given that transposases in many cases are recruited to particular genomic niches via physical interaction with pre-seated proteins (16,17,23–27), a tethering model has been proposed for TE targeting (10,26,27). In addition, the timing of transposition is critical for copy number increase of cut-and-paste TE element and is therefore important for TE amplification. For instance, transposition of *Activator* primarily takes place after replication of the donor site but before replication of the target site, resulting in a net increase in copy number following cell replication (28).

Although there has been a great deal of analysis of *de novo* insertions of TEs in a variety of species, there have been few broad comparisons of TE insertion profiles in both animals and plants. Gene expression is often associated with open, accessible chromatin, which in turn is associated with insertion of a number of elements in both plants and animals (12,13). Further, RNAseq gene expression data are available from a broad range of tissues in multiple species, making direct comparisons relatively easy (29–31). With this in mind, we performed a comparison of multiple TEs in multiple species and examined TE distribution in the context of genome-wide transcriptional landscapes using multiple relatively unselected *de novo* transposon insertion datasets collected by many groups, including our own. We identify two distinct types of Pol II-associated TE targeting strategies, as well as those that are independent of Pol II transcription, and we provide evidence for convergent evolution among plant and animal TE families. We also provide data that suggests that TEs have evolved strategies to minimize their effects on host gene expression, even when those TEs specifically target gene space.

## MATERIALS AND METHODS

### Collecting *de novo* transposon coordinates and RNA-seq datasets

The TE families that were analyzed in this study have all been employed as efficient mutagens and each has been used for sequence-indexed mutant library construction. The UniformMu, *Dissociation* (*Ds*)*-GFP*, *Transposon of Oryza sativa 17* (*Tos17*), *Ds*, *Suppressor-mutator* (*Spm*), *P*, *piggyBac* (*Pb*) and *Minos* (*Mb*) *de novo* transposon insertion collections in maize, rice and *Drosophila* are publicly available. Coordinates of TE insertions were retrieved from relevant websites and databases (32–38). Somatic *Mu* elements (SomaticMu elements) have been generated by performing *Mu*-seq with leaves collected from high-copy *Mu*-active maize seedlings and coordinates were called using the same pipeline as was used for the germinally inserted UniformMu collection (39). In the >320,000 SomaticMu insertions analyzed, a few hundred germinal insertions (ancient and germinally transmitted background insertions) were not removed as their impact on the overall distribution of SomaticMu insertions is neglectable. We also collected 1358 annotated Pack-MULEs in the maize genome (ftp://ftp.gramene.org/pub/gramene/release61/gff3/zea_mays/repeat_annotation/B73v4.TE.filtered.gff3.gz) and 2959 Pack-MULEs in the rice genome from the literature (40). The TE coordinates in each organism were made consistent with current genome assembly versions (AGPv4 B73 for maize, Oryza sativa.IRGSP-1.0.42 for rice and FB2014_03, R5.57 for fly). All coordinates for all insertions in each species are provided in Supplemental Tables S1–S5. Coordinates for insertions near tRNA and rRNA genes are provided in Supplemental Table S6.

Raw FPKM (Fragments Per Kilobase of transcript per Million mapped reads) values of publicly available RNA-sequencing experiments for AGPv4 genes from maize were retrieved from the Maize Genetic Resource database (http://maize.plantbiology.msu.edu) (29). PCA analysis was performed based on the average FPKM values generated from RNAseq datasets of different maize tissues using the R package FactoMineR (41). The rice and *Drosophila* RNAseq data were retrieved from the Rice Expression Database and FlyAtlas 2, respectively (30,31). Expression levels for all genes used this analysis are available in Supplemental Table S7.

### Meta-analysis of transposon distributions near transcription start sites (TSSs) and transcription termination sites (TTSs) of genes at various transcriptional landscapes

For a given gene set (all genes in a genome or a gene subset), each *de novo* and ancient TE insertion was classified as being either genic or intergenic and distances from transcriptional start sites (TSSs) and transcriptional termination sites (TTSs) were calculated for each insertion event. Genic insertions were plotted along the positive X-axis and intergenic insertions were plotted along the negative X-axis relative to the TSS and TTS in comparison to randomly selected genomic loci. A total 421 280 random insertions were *in-silico* generated on the maize chromosomes at a density of one insertion per 5 kb, which is comparable to the largest insertion dataset (SomaticMu). The densities of random insertions in both rice and Drosophila were set to be one insertion per 400 bp on average given that these genomes are relatively small and gene-rich. For each TE insertion or randomly selected locus within intergenic regions, its distance to the TSS and the TTS of both the nearest upstream gene and the nearest downstream gene were counted in the meta-profiling plots. In a number of gene rich regions, a small proportion of TE insertions or randomly distributed loci are <4 kb from both upstream and downstream genes, so they were counted twice. This would be expected to cause a mildly uneven distribution of a subset of the random selected loci.

The metaprofiles for intergenic and genic transposon insertions were plotted separately, using normalized insertion numbers in sliding 30-basepair (bp) windows centered on each position. In order to compare the enrichment of TEs near all annotated genes (or subsets of genes), normalization of the insertion numbers was performed by calculating the number of insertions per 30 bp window per 100 000 insertions per 10 000 genes at each position surrounding TSSs or TTSs. For both the TSSs and TTSs, genic transposon insertions were plotted along the positive X-axis while the intergenic insertions were plotted along negative X-axis.

For CHH islands near the 5′ or 3′ ends of genes, the relative position of these islands (which are 100 bp in length) and gene TSSs or TTSs was unified in such a way that the CHH islands were located on an interval [−50, 49] on X-axis of each plot, and adjacent genes were placed downstream of both 5′-end CHH and 3′-end CHH on the positive X-axis. Insertion numbers at each position are normalized to 100 000 insertions and 10 000 CHH islands. Given the repetitive nature of tRNA, and particularly rRNA genes, special care was taken to ensure that only independent insertions were counted by using polymorphisms between sequences flanking the insertions in these genes. The ∼30% of insertions into tRNA and rRNA genes that lacked sufficient polymorphism were not included in our analysis.

Independent RNAseq experiments were treated as replicates in each organism. For each experiment, genes were placed into 20 bins based on their relative level of expression, with bin 1 representing the lowest level of expression and bin 20, the highest. The percentage of TSS-associated insertions (<2 kb upstream of TSSs) in each bin were calculated for each RNAseq dataset, and the averaged percentages in all datasets were plotted along the X-axis. For each experiment, a bin represents a categorical level of gene expression. That is to say, a bin is not always the collection of identical genes in independent RNAseq experiments, but rather it contains a set of genes whose ranks based on their expression level fall in the same category in each particular experiment.

### Sequencing-based transposon profiling and sequencing-based allele frequency analysis

Miseq-based *Mu* element profiling was performed as described previously using F1 hybrid progeny seedlings (42). The B73 parent was carried *Mutator* activity that had been introgressed into the B73 genetic background. The Mo17 parent lacked active *Mu* elements. Thus, all new insertions were into the B73 genome. Genomic DNA was extracted from 6-day-old seedlings of B73/Mo17 hybrid plants. Amplicon-based enrichment of *Mu* flanking DNA was then performed. The purified PCR products were subject to Miseq-based Wideseq pipeline at Purdue Genomics Core Facility (https://www.purdue.edu/hla/sites/genomics/wideseq-2/). Wideseq reads were mapped to the B73 reference genome as described previously (42). By identifying the *Mu* target site duplications (TSDs), a set of genes targeted by *Mu* insertions that segregated in hybrid progeny was obtained and those containing B73/Mo17 SNPs in their mRNA sequences were used for allele-specific expression analysis. Because new insertions were into the B73 genome, the effect of these insertions would be expected to be specific in all cases to the B73 allele. To quantify the allele frequency, we performed RT-PCR followed by Wideseq from the identical shoot tissues of the hybrid seedlings mentioned above. Total RNA was extracted using the RNA Extraction Kit (Zymo) and cDNAs were synthesized using Promega M-MLV Reverse Transcriptase. For a subset of 16 genes that carried SNPs, RNA fragments containing B73/Mo17 SNPs were amplified by RT-PCR. Primers used for this analysis are provided in Supplemental Table S8. The RT-PCR products were then sequenced by the ‘WideSeq’ pipeline. We also performed RNAseq on endosperms of four individual hybrid seeds. Preliminary processing of RNAseq reads and transcriptome mapping were carried out as described previously (43). As above, segregating *Mu* insertions were identified using Wideseq in these four hybrid individuals. For 16 genes with segregating *Mu* insertions, SNPs are available so that the allele frequency in the B73/Mo17 hybrid transcripts could be called. Fold changes of gene expression caused by *Mu* insertions for each gene were calculated by comparing the B73 allele frequency in individuals containing *Mu* insertions with those without *Mu* insertions which were further normalized using the Mo17 allele transcript frequency in plants that lacked an insertion in either B73 or Mo17.

## RESULTS

### Distribution of *de novo* transposons near TSSs and TTSs of host genes

To understand *bona fide* target preferences of transposons, we examined the target site distribution of nine *de novo* insertion datasets. These included the UniformMu (44), SomaticMu (this report) and *Ds-GFP* collections in maize (32,33), the *Tos17*, *Ds* and *Spm* collections in rice (34–36), as well as the *P-*element, *Pb* and *Mb* insertion collections in *Drosophila* (37,38). All TEs examined here are DNA transposons with the exception of *Tos17*, which is a low copy number LTR retrotransposon. UniformMu is primarily composed of germinally transmitted *Mu* insertions and SomaticMu elements are inferred to be derived primarily from somatic insertions due to the relative low number of reads obtained relative to the insertions that segregated in the families examined.

**Figure 1.**
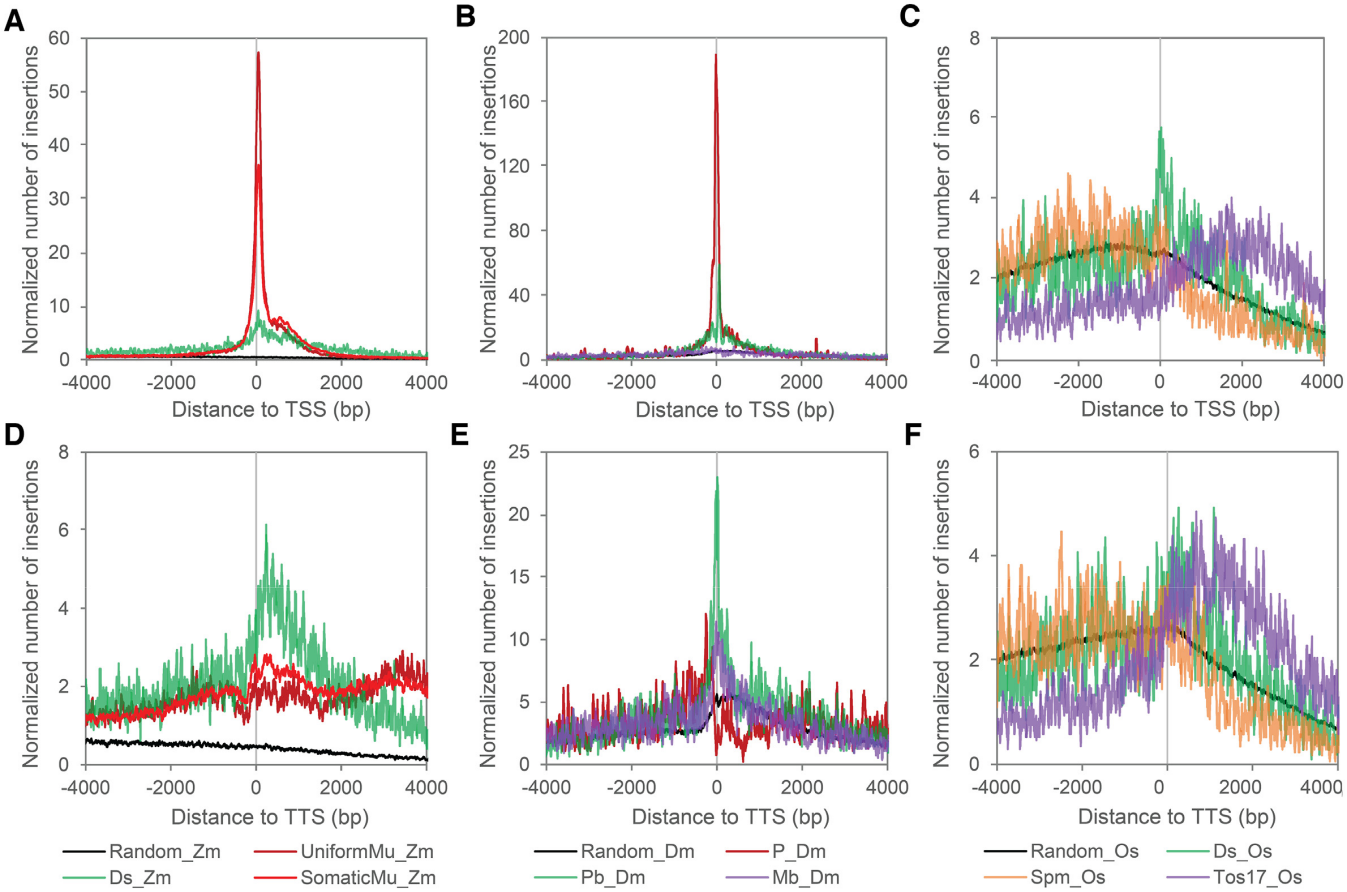
Characterization of distribution of *de novo* transposon insertions that are near genes. The figure shows normalized numbers of insertions around TSSs and TTSs of annotated genes. The insertion numbers at each distance was smoothed by computing the mean in a 30-bp rolling window. For both TSS and TTS plots, normalized numbers of genic insertions were plotted on the positive X-axis coordinates and normalized numbers of intergenic insertions were plotted on the negative coordinates. Thus, positive values for both plots represent insertions into the gene body. Randomly selected loci were used as a background control to take into account the fact that although insertions were plotted up to 4 kilobase pairs (kb) away from the TSSs and TTSs, many gene bodies are shorter than 4 kb, and many genes are more or less than 4 kb downstream or downstream from a neighboring gene. Panels in the upper and bottom rows show metaprofiles of transposon insertions in maize (**A, D**), Drosophila (**B, E**) and rice (**C, F**) for regions surrounding the gene TSS and TTS, respectively.

A comparative analysis of insertion profiles relative to randomly selected loci revealed dramatic similarities and differences between different elements in different species with respect to their association with TSSs or TTSs. The difference between the distribution pattern of TE insertions and that of randomly selected loci reveals a dramatic enrichment of TE insertions near TSSs or TTSs for some elements. Both the *Mu* element (UniformMu and SomaticMu) and *P* element insertions were vastly enriched near TSSs (peak shift < 50 bp), but were largely missing near TTSs, indicating a tight TSS-specific association with both transposases (Figure 1A, B, D, E, Supplementary Figure S1A, B, D, E). In addition, the distribution curves of *Mu* element insertions decrease rapidly upstream of the TSSs but are reduced more gradually downstream of the TSSs, particularly within 1 kb (Supplementary Figure S1A). Given the extreme bias in integration of *Mu* and *P* elements, we refer to these elements as precision-targeting elements. In contrast, enrichment of *Ds* and *Pb* insertions was observed near both the TSSs and TTSs, with much wider and lower peaks, suggesting less specificity than *Mu* and *P* elements (Figure 1A, B, D, E, Supplementary Figure S1A, B, D, E). There is no enrichment of *Mb*, *Spm* and *Tos17* insertions near either TSSs or TTSs. Indeed, *Tos17* insertions are actually somewhat enriched in the gene body relative to these sites (Figure 1C, F, Supplementary Figure S1C, F).

**Figure 2.**
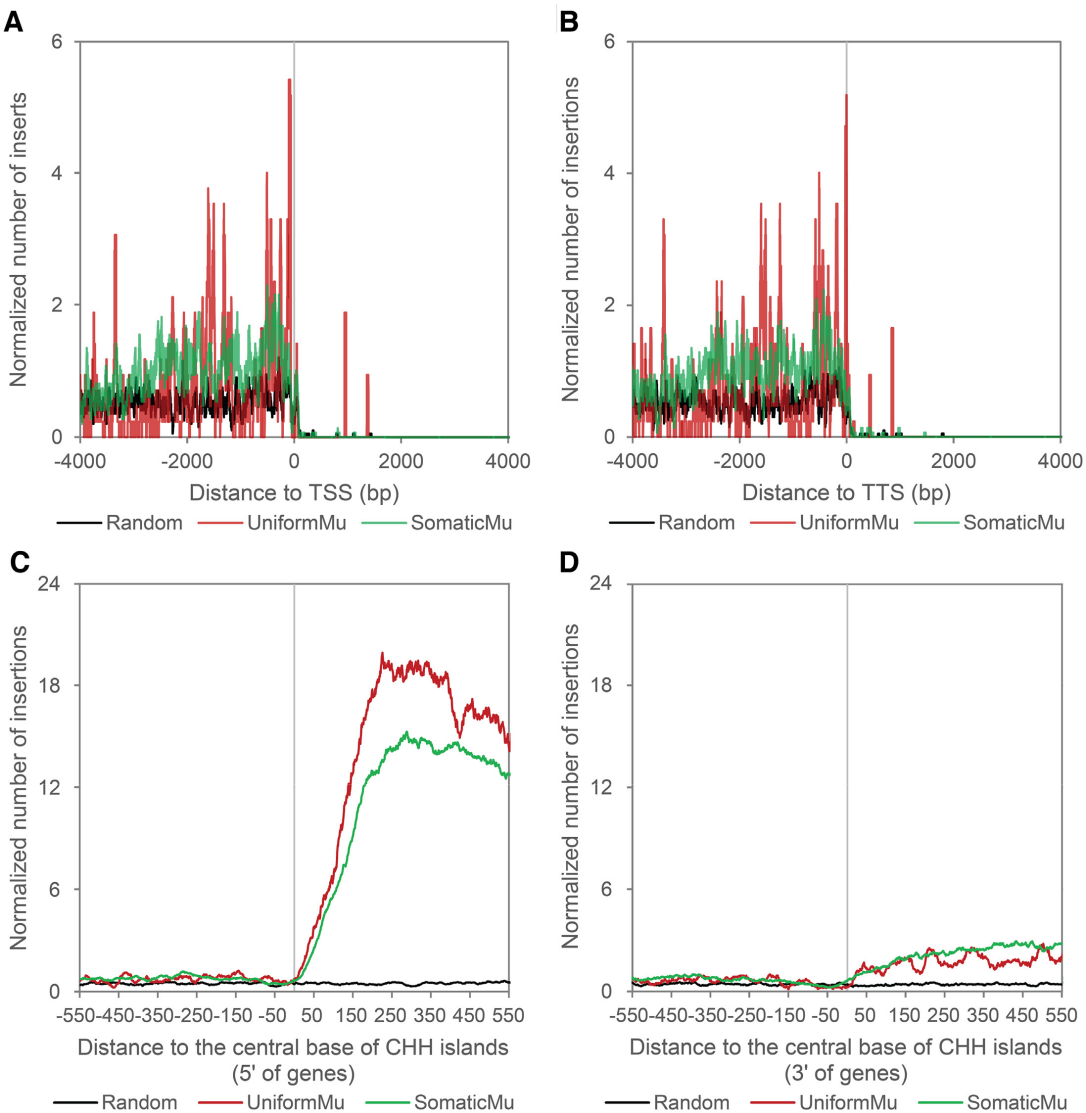
Distribution of *de novo Mu* insertions near target sites of RNA Polymerases I, III, IV and V. (A and B) Metaprofiles of UniformMu and SomaticMu insertions surrounding TSS (**A**) and TTS (**B**) of tRNA and rRNA genes. The figure shows normalized numbers of insertions around TSSs and TTSs of annotated tRNA and rRNA genes. The insertion numbers at each distance were smoothed by computing the means in 30-bp rolling windows. Normalized numbers of genic insertions were plotted on the positive X-axis coordinates and those of intergenic insertions were plotted on the negative coordinates. (C and D) Distribution of UniformMu and SomaticMu insertions near CHH islands in maize located at 5′ (**C**) and 3′ ends (**D**) of maize genes. The 100-bp CHH islands are centered on position zero and range from −50 to 49 bp on the X-axis. Directions of CHH islands were unified so that nearby genes are located on the right of both 5′ and 3′ CHH islands.

To determine whether *Mu* and *P* element targeting is specific to Pol II-dependent transcription or is actually associated with any RNA polymerase, we examined the distribution of *de novo Mu* element and *P* element insertions in maize and *Drosophila*, respectively, near rRNA and tRNA genes, which are transcribed by RNA Pol I or III, respectively. To minimize Pol II TSS-associated TE enrichment, we filtered the rRNA and tRNA gene set in maize based on their distance to Pol II TSSs and obtained a list of 1610 genes over 5 kb away from the TSSs of any annotated genes transcribed by Pol II. Few transposon insertions are into the gene body of the 1610 rRNA and tRNA genes, as can be observed along the positive X-axis, likely due to the small size of these genes. In regions both downstream of TTSs and upstream of TSSs of these genes, UniformMu and SomaticMu resemble random selected genomic loci in their distribution except that the UniformMu shows a less smooth distribution curve than the SomaticMu (Figure 2A, B), which can be attributed to the fact that there are ∼3.5 times as many SomaticMu insertions as there are UniformMu insertions. Similar to *Mu* element insertions, *P* elements insert into or near the TSSs and TTSs of rRNA and tRNA genes at a frequency comparable to randomly selected loci (Supplementary Figure S2).

Plants have two plant-specific RNA polymerases, Pol IV and Pol V, that are required for cytosine methylation in asymmetrical (CHH, where H is A, T or C) sequence contexts (45). A large number of CHH islands are located immediately upstream of the 5′ ends or downstream of the 3′ ends of genes in maize (46). We observed no enrichment of *Mu* element insertions in CHH islands; the vast majority of insertions are adjacent to the 5′ CHH islands, where Pol II transcripts are initiated at TSSs. These results indicate that Pol IV and Pol V transcription start or stop sites are not notable targets for *Mu* element insertions (Figure 2C, D).

### Distribution of TE target sites near host genes with different expression levels

**Figure 3.**
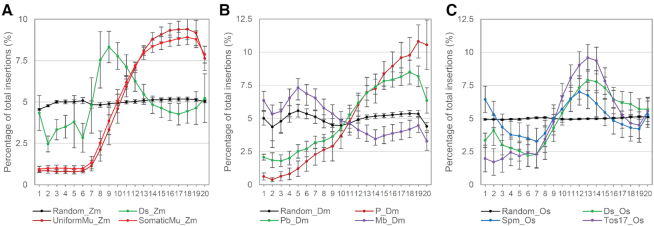
*De novo* transposon insertions near genes expressed at different levels. Percentage of transposon insertions distributed in each of the 20 expression bins were shown for maize (**A**), Drosophila (**B**) and rice (**C**) transposons. The figures show the correlation between TE targeting frequency with categorized gene expression levels. For each RNA-seq dataset, the whole annotated gene set was grouped to 20 expression bins from low to high independently, each containing an equal number of genes. Allocation of genes in each bin was independently performed for each RNA-seq experiment. Each RNA-seq dataset was regarded as one replicate and the averaged percentages in each categorized expression levels were calculated. Error bars represent standard deviations of the percentages in each bin based on independent RNAseq datasets.

Given that the target sites of several TE families examined are TSS- or TTS-associated, we hypothesized that the transposases of some families are recruited to TSSs or TTSs in a manner that is dependent on the level of transcription. To test this hypothesis, we examined the correlation between transposon targeting frequency and relative expression levels of host genes. We extracted a subset of TSS-associated TE insertions that are located near (<2 kb) the TSSs and retrieved publicly available RNAseq datasets from 79 tissues in maize, 38 tissues in *Drosophila* and 59 tissues in rice (29–31). For each dataset, genes were binned into 20 equal sized groups based on ranked FPKM values in each RNAseq experiment, where bin 1 contains the lowest expressed 5% genes and bin 20 contains the highest expressed 5% genes. While randomly selected genomic loci (the control datasets) were evenly distributed near genes expressed at various levels, *Mu* and *P* elements preferentially target highly expressed genes, as indicated by the upward sloping curves (Figure 3A, B). Targeting frequency of *Pb* also positively correlates with gene expression, but to a lesser extent than that of *P* and *Mu* elements (Figure 3B). Interestingly, the distribution of *Ds* (in both maize and rice), *Spm* and *Tos17* transposon insertion hotspots are all overrepresented in the medium expression bins (Figure 3A, C), suggesting that genes expressed at these levels are preferred targets for these elements. In contrast, the *Mb* elements, which show a mild enrichment at TTSs but not TSSs, actually target the lowest expression bins (Figure 3B).

Using the two available *Mu* insertion datasets, we tested the hypothesis that genes frequently targeted by precision-targeting TEs are associated with specialized biological functions or processes by performing gene ontology (GO) analysis of 4225 *Mu* element hotspot genes (>3 UniformMu and >10 SomaticMu insertions <2 kb from TSSs, Supplementary Table S1) using the online webserver agriGO (47). The seven GO terms enriched for *Mu* hotspot genes are related to a wide range of general, rather than specialized, biological processes and molecular functions (Supplementary Figure S3A). Genes categorized by these seven GO terms, on average, were expressed at markedly higher levels than the total gene set (Kolmogorov-Smirnov test, *P* values as indicated next to the box plots) (Supplementary Figure S3B), consistent with our observation that *Mu* elements preferentially target highly expressed genes. Moreover, in each of these seven gene sets, *Mu* hotspot genes are expressed at a significantly higher level than the non-hotspot genes in each set (Kolmogorov–Smirnov test) (Supplementary Figure S3C). These observations suggest that these GO terms are enriched not because they are associated with particular processes, but because they tend to express at higher levels than the average gene.

### Distribution of TE insertions in meristematic and differentiated tissues

Given that the targeting frequency of several TE families associates with host gene expression levels in a collection of tissues, we hypothesized that tissues showing the strongest correlation between the two would be the tissues in which transposition occurs most frequently, assuming transposition has tissue-specificity. We explored the tissue specificity of TE transposition by using *Mu* and *Ds* elements in maize as examples.

**Figure 4.**
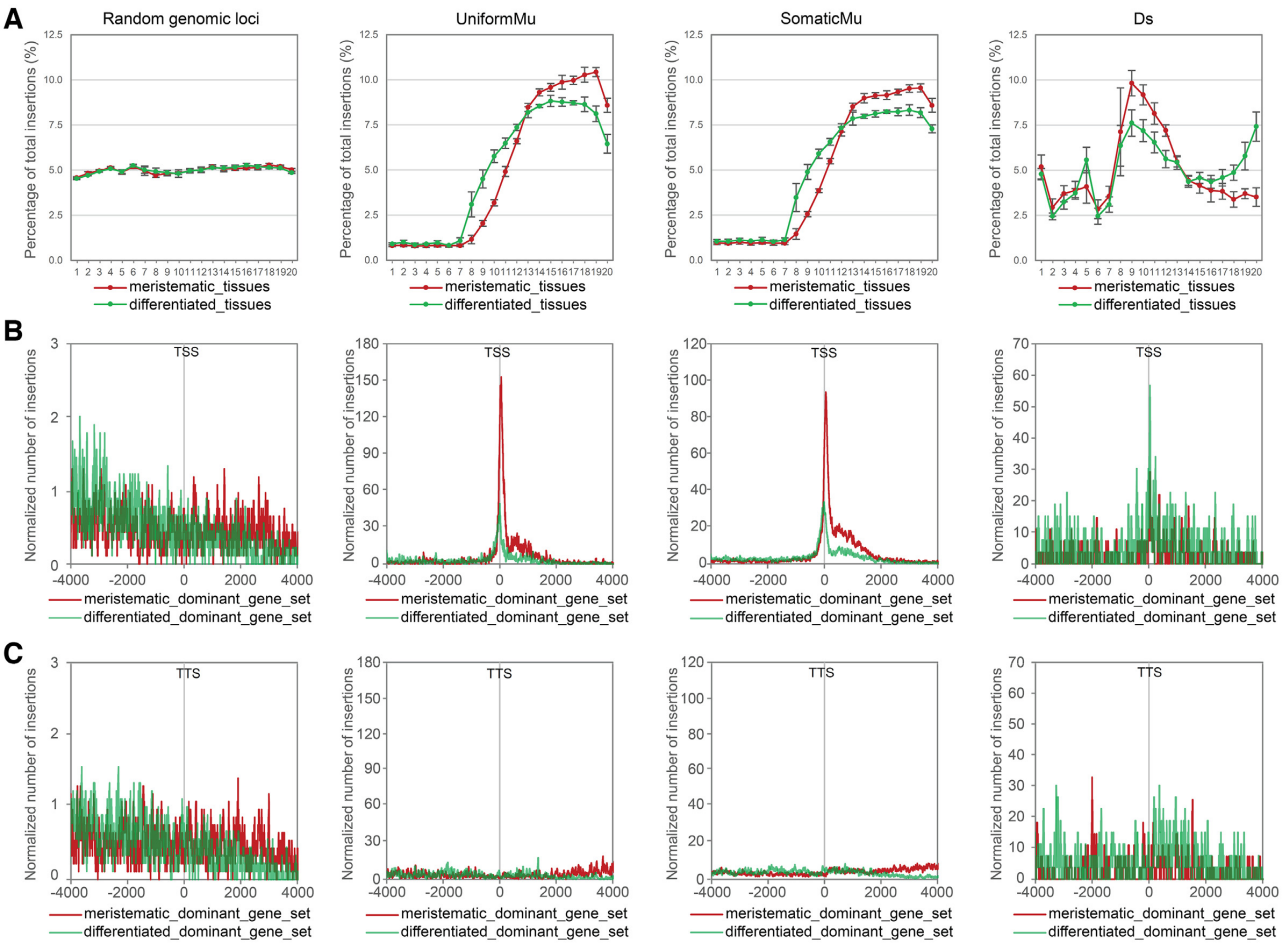
Comparative analysis of the distribution of *Mu* and *Ds* elements in meristematic or differentiated tissues. (**A**) Percentage of random genomic loci, UniformMu, SomaticMu and *Ds* element insertions distributed in each of 20 expression bins in meristematic and differentiated tissues. An identical set of randomly selected loci that was used in Figure 1A (and 1D) was used as a background control. Error bars represent standard deviations of the percentages in each bin based on independent RNAseq datasets. (B and C) Metaprofiles of random genomic loci, UniformMu, SomaticMu and *Ds* elements in maize surrounding TSSs (**B**) and TTSs (**C**) of meristematic-dominant and differentiated-dominant gene sets, respectively. For both TSS and TTS plots, normalized numbers of genic insertions were plotted on the positive X-axis coordinates and normalized numbers of intergenic insertions were plotted on the negative coordinates. The insertion numbers at each distance were smoothed by computing the means in 30-bp rolling windows. An identical set of randomly selected loci used in Figure 1A (and 1D) was used as a background control.

To explore the major factors that contribute to transcriptome variation in different maize tissues, we first performed principle component analysis (PCA) on the above-mentioned 79 RNAseq datasets and found that the first principle component (17.8% of the variance) separated tissues of meristematic and differentiated identities well (Supplementary Figure S4A). The distribution curves of *Mu* and *Ds* element insertions in low-to-high expression bins showed distinct patterns in the six meristematic and six differentiated tissues (Figure 4A, Supplementary Figure S4B). The two curves differed most in the medium (bins 8–12, ranked between 35% and 60%) and highly (bins 16–20, ranked between 75% and 100%) expressed genes (Figure 4A). To address whether tissue-specific gene expression in meristematic and differentiated tissues associated with this shift of the distribution curve, we identified genes that were expressed at high levels in the meristem-enriched tissues and at medium levels in the differentiated tissues (meristematic-dominant genes), and those that were expressed at high levels in differentiated tissues and medium levels in meristem enriched tissues (differentiated-dominant genes). We obtained a set of 746 meristematic-dominant genes that were present in bins 16–20 in the majority (no less than five) of six meristematic tissues and in bins 8–12 in the majority (no less than five) of six differentiated tissues. We also obtained a set of 723 differentiated-dominant genes that were present in bins 16–20 in the majority (no less than five) of six differentiated tissues and in bins 8–12 in the majority (no less than five) of six meristematic tissues (Supplementary Figure S5). We found a much higher enrichment of *Mu* element insertions (both UniformMu and SomaticMu) that were near TSSs of the meristematic-dominant genes than were near TSSs of differentiated-dominant genes (Figure 4B). Such enrichment is specific to TSSs, but not TTSs, consistent with our previous observations (Figure 4C). We also found that *Ds* enrichment in the medium expression bins 8–12 is higher in meristematic tissues (Figure 4A), likely due to a preference for medium expressed genes by *Ds* elements. In line with this observation, we observed a lower level of *Ds* enrichment near TSSs, and to a lesser extent, TTSs in the meristematic-dominant gene set (Figure 4B, C), suggesting that *Ds* elements insert at a higher frequency in genes that express at a medium level in meristematic tissues (the differented-dominant gene set). These results suggest that both *Mu* and *Ds* elements insert most frequently in genes that express at targeted levels in meristematic or rapidly dividing cells. In the case of *Mu*, these are genes that express at a high level in those cells. In the case of *Ds*, it is genes that express at a medium level in those cells.

### Re-evaluation of the mutagenic capability of precision-targeting transposons

TE insertions in regions in or near genes may or may not disrupt host genes depending on where the TEs integrate. To evaluate the mutagenic capability of the precision-targeting transposons, we examined the enrichment of *Mu* and *P* elements in 5′ and 3′ proximal regions as well as sub-genic features of annotated maize and fly genes, respectively. Consistent with the meta-analysis above (Figure 1), insertions of both *Mu* and *P* element show strong enrichment in the 5′ ends of genes (particularly 200 bp upstream of the TSSs and 5′ UTRs) (Table 1). *Mu* element insertions are enriched in 5′ UTRs (64.4-fold for *SomaticMu*; 90.1-fold for *UniformMu*) relative to insertions into coding sequences (8.8-fold for *SomaticMu*; 7.0-fold for *UniformMu*) (all *P* values less than 1E−5, χ^2^ test). Enrichment of *P* elements at the 5′ ends of genes is coupled with a 4-fold lower frequency of *P* elements insertions into coding sequences relative to random chance. Indeed, out of a total of *P* element 18 213 insertions, only 729, or 4%, are inserted into coding sequences (CDSs).

**Table 1.** A summary of the numbers and percentages of *Mu* and *P* elements insertions in or near genes. For random insertions, enrichment fold is defined as 1; values above 1 mean an enrichment of TE insertions, values below 1 mean a depletion of TE insertions

	Total (100%)	Genic				500 bp upstream of TSS				200 bp upstream of TSS				5′ UTR			
	Count	Count	% of total	Enrichment fold	*P*-value (χ² test)	Count	% of total	Enrichment fold	*P*-value (χ² test)	Count	% of total	Enrichment fold	*P*-value (χ² test)	Count	% of total	enrichment fold	*P*-value (χ² test)
Random_Zm	421280	32246	7.65%	1	NA	3985	0.95%	1	NA	1544	0.37%	1	NA	1268	0.30%	1	NA
UniformMu_Zm	92702	54040	58.29%	7.62	<1E−5	15976	17.23%	18.22	<1E−5	12103	13.06%	35.62	<1E−5	25142	27.12%	90.11	<1E−5
SomaticMu_Zm	320044	181776	56.80%	7.42	<1E−5	45582	14.24%	15.06	<1E−5	32739	10.23%	27.91	<1E−5	62028	19.38%	64.39	<1E−5
Pack_MULE_Zm	1369	183	13.37%	1.75	<1E−5	167	12.20%	12.9	<1E−5	94	6.87%	18.73	<1E−5	20	1.46%	4.85	<1E−5
Random_Dm	349280	222916	63.82%	1	NA	13746	3.94%	1	NA	6511	1.86%	1	NA	11315	3.24%	1	NA
P_Dm	18213	12586	69.10%	1.08	<1E−5	5485	30.12%	7.65	<1E−5	4409	24.21%	12.99	<1E−5	4578	25.14%	7.76	<1E−5
	Total (100%)	CDS				3'UTR				200 bp downstream of TTS				500 bp downstream of TTS			
	Count	Count	% of total	Enrichment fold	*P*-value (χ² test)	Count	% of total	Enrichment fold	*P*-value (χ² test)	Count	% of total	Enrichment fold	*P*-value (χ² test)	Count	% of total	Enrichment fold	*P*-value (χ² test)
Random_Zm	421280	9188	2.18%	1	NA	1889	0.45%	1	NA	1508	0.36%	1	NA	3781	0.90%	1	NA
UniformMu_Zm	92702	14220	15.34%	7.03	< 1E-5	1471	1.59%	3.54	< 1E-5	1220	1.32%	3.68	<1E−5	2703	2.92%	3.25	<1E−5
SomaticMu_Zm	320044	61393	19.18%	8.8	< 1E-5	7046	2.20%	4.91	< 1E-5	5760	1.80%	5.03	<1E−5	12279	3.84%	4.27	<1E−5
Pack_MULE_Zm	1369	17	1.24%	0.57	0.01947	25	1.83%	4.07	< 1E-5	16	1.17%	3.27	<1E−5	52	3.80%	4.23	<1E−5
Random_Dm	349280	56979	16.31%	1	NA	18468	5.29%	1	NA	5498	1.57%	1	NA	10968	3.14%	1	NA
P_Dm	18213	729	4.00%	0.25	< 1E-5	264	1.45%	0.27	< 1E-5	333	1.83%	1.16	0.00849	967	5.31%	1.69	<1E−5

Assuming that TE insertion into 5′ end of genes are less deleterious than CDS insertions, we further examined the degree to which insertions of precision targeting TEs affect the expression of nearby genes. To do this, we evaluated the consequences of a list of *de novo Mu* element insertions, most of which are into promoter or 5′ UTRs, by experimentally testing the fold change of gene expression levels caused by *Mu* element insertions. A *Mu*-active maize line in the B73 background was crossed with a *Mu*-inactive line in the Mo17 background. The segregating *Mu* element insertions in the progeny were profiled using a Miseq-based amplicon-sequencing pipeline (42) and the B73-Mo17 SNPs were called for quantifying relative transcript levels of both parental alleles with and without *Mu* element insertions using deep sequencing (Supplementary Figure S6). A knockdown index was deduced by normalizing the observed ratio to that observed in genes that lacked *Mu* insertions in both genetic backgrounds for each insertion. We found that none of the four promoter insertions changed the expression of nearby genes. A quarter of 5′ UTR insertions (5 out of 20) caused knockout or strong knockdown effects and one of seven intronic insertions resulted in a knockout effect (Table 2). Collectively, of a total of 33 *Mu* insertions, all of which were within 200 bp of genes, only 11 significantly reduced gene expression, and only two eliminated completely expression. These results indicate that *Mu* element insertions near TSSs of host genes are often associated with quantitative and in many cases neglectable functional consequences on nearby gene expression.

**Table 2.** Re-evaluation of mutagenic capability of *Mu* insertions by RNAseq and sequencing RT-PCR products via Miseq

Affected_gene	Insertion_site	B73_insertion	Mo17_no_insertion	B73_no_insertion	Mo17_no_insertion	Knockdown_index	**Consequence**
Zm00001d020901	five_prime_UTR	0	247	461	519	∞	**knockout**
Zm00001d027950	intron	2	517	221	285	200.5	**knockout**
Zm00001d039733	five_prime_UTR	39	960	553	480	28.4	**knockdown**
Zm00001d044446	five_prime_UTR	31	703	148	144	23.3	**knockdown**
Zm00001d028712	five_prime_UTR	270	510	192	30	12.1	**knockdown**
Zm00001d006460	five_prime_UTR	86	102	50	5	11.9	**knockdown**
Zm00001d017424	five_prime_UTR	6	34	819	1065	4.4	**weak_knockdown**
Zm00001d005587	five_prime_UTR	10	60	81	122	4	**weak_knockdown**
Zm00001d023962	five_prime_UTR	78	488	181	463	2.4	**weak_knockdown**
Zm00001d008642	five_prime_UTR	119	1235	361	1782	2.1	**weak_knockdown**
Zm00001d006126	intron	83	68	144	50	2.4	**weak_knockdown**
Zm00001d006768	promoter -65	63	93	79	62	1.9	**unchanged**
Zm00001d033167	promoter-117	154	69	140	112	0.6	**unchanged**
Zm00001d039683	promoter-32	206	95	166	86	0.9	**unchanged**
Zm00001d012812	promoter-78	752	405	239	138	0.9	**unchanged**
Zm00001d039156	five_prime_UTR	455	838	542	676	1.5	**unchanged**
Zm00001d047761	five_prime_UTR	161	393	50	83	1.5	**unchanged**
Zm00001d018461	five_prime_UTR	402	818	223	383	1.2	**unchanged**
Zm00001d022153	five_prime_UTR	320	208	122	79	1	**unchanged**
Zm00001d034667	five_prime_UTR	924	854	288	302	0.9	**unchanged**
Zm00001d034191	five_prime_UTR	138	96	36	30	0.8	**unchanged**
Zm00001d028784	five_prime_UTR	27	20	355	349	0.8	**unchanged**
Zm00001d050081	five_prime_UTR	53	53	31	42	0.7	**unchanged**
Zm00001d039253	five_prime_UTR	30	184	16	133	0.7	**unchanged**
Zm00001d001788	five_prime_UTR	233	178	497	563	0.7	**unchanged**
Zm00001d029856	intron	189	314	104	96	1.8	**unchanged**
Zm00001d050163	intron	8	11	127	110	1.6	**unchanged**
Zm00001d053452	intron	963	1324	236	310	1	**unchanged**
Zm00001d049619	intron	70	260	251	1275	0.7	**unchanged**
Zm00001d006610	three_prime_UTR	336	292	288	232	1.1	**unchanged**
Zm00001d029059	five_prime_UTR	244	131	46	59	0.4	**unchanged/weak_activation**
Zm00001d022122	intron	43	29	2	9	0.1	**unchanged/weak_activation**

**Table 3.** Classification of integration strategies used by TE families analyzed in this study based on their distribution near genes and the association of TE targeting frequency with gene expression levels

Strategy	Typical TE family	Host	Associated region	Expression levels of target genes
A	*Mu*	Maize	TSS	High
A	*P*	Drosophila	TSS	High
B	*Ac/Ds*	Maize, rice	TSS and TTS	Medium-to-high
B	*Piggybac*	Drosophila	TSS and TTS	Higher than *Ac*/*Ds*, lower than *Mu* and *P*
C	*Minos*	Drosophila	Not observed	Low-to-medium
C	*Tnt1*	Medicago	Gene body	Low-to-medium
C	*Tos17*	Rice	Gene body	Medium-to-high
C	*Spm*	Rice	Not observed	Medium-to-high

**Figure 5.**
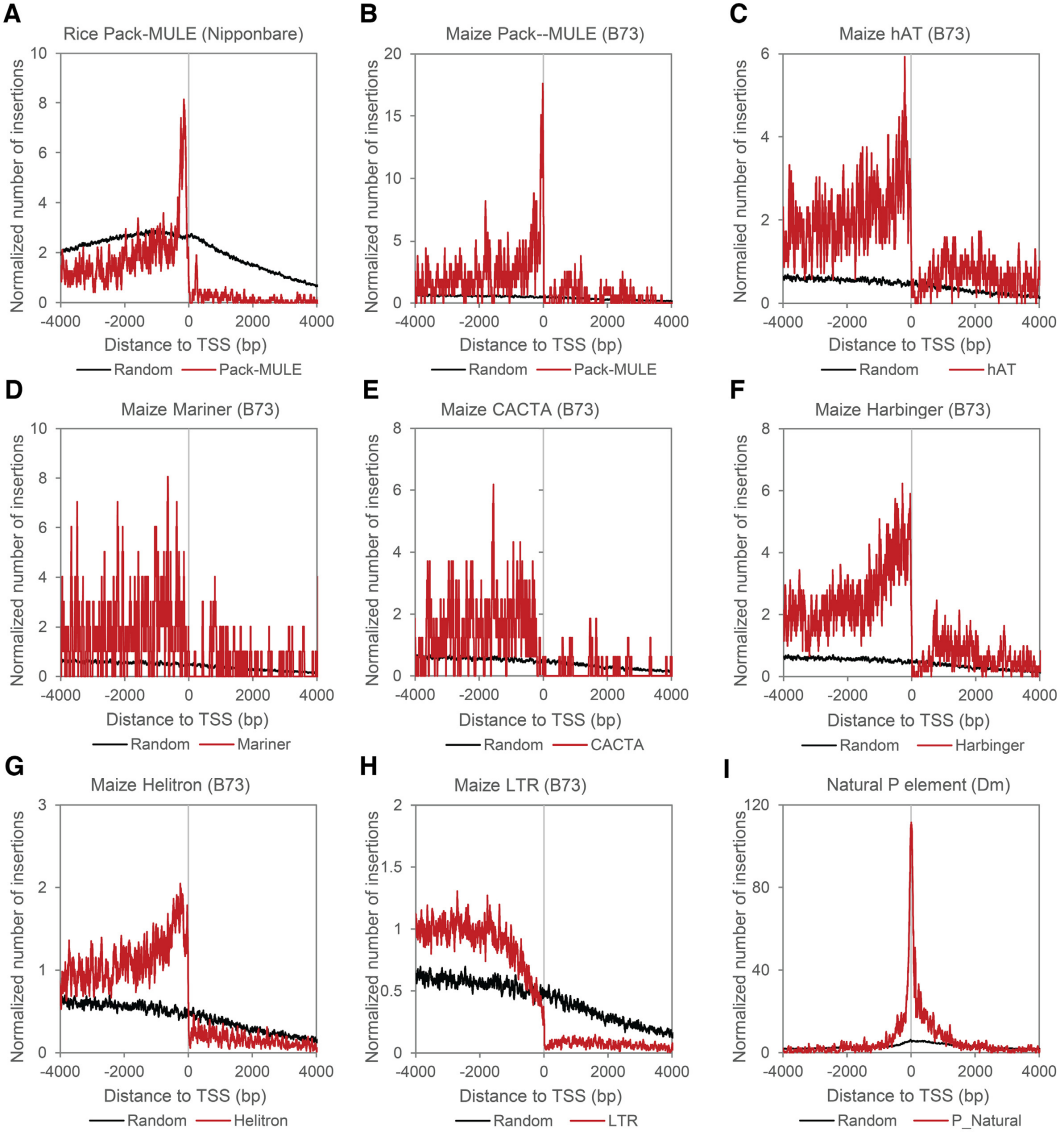
Metaprofiles of ancient transposons near the TSS of host genes. Normalized numbers of insertions around TSSs and TTSs of annotated genes. Genic insertions were plotted on the positive X-axis coordinates and intergenic insertions were plotted on the negative coordinates. The insertion numbers at each distance were smoothed by computing the means in 30-bp rolling windows. Identical sets of randomly selected loci used in Figure 1 were used as a background control.

Over a longer time-scale, purifying selection would be expected to purge insertion mutations that have only weak deleterious effects. To evaluate the selection pressures on older *Mu* element insertions, we examined a class of *Mutator*-like elements called Pack-MULEs, many of which are ancient insertions in the genomes that have diverged terminal inverted repeats (TIRs) (48). Profiling the distribution of 1358 Pack-MULEs in maize and 2959 Pack-MULEs in rice surrounding TSSs show that in both species, Pack-MULE occupancy peaks just upstream of TSSs and is reduced to nearly background levels >1 kb upstream of TSSs (Figure 5A, B). This is quite similar to our observation of *de novo* insertions in maize. However, there is a sharp decline of Pack-MULE insertions into gene bodies (Figure 5A, B), indicating selection pressure against older genic insertions into this region. Consistent with this observation, and consistent for selection against insertion into genes, underrepresentation of genic transposon insertions downstream of TSSs were found in other DNA transposon families in the maize genome, including hAT, Mariner, CACTA, Harbinger and Helitrons and LTR retrotransposons (Figure 5C-H). In contrast, we found that *P* element annotated in the genomes of wild *D. melanogaster* accessions exhibit an identical distribution of the *de novo P* element insertions (Figures 1B and 5I). Presumably, this is due to the fact that *P* elements have only been in the *D. melanogaster* genome for a relatively short period of time and are unlikely to be fixed or homozygous in wild populations (49).

## DISCUSSION

It has been suggested that genomes resemble ecological systems, and that different TE families occupy distinct niches, presumably because there are multiple ways to be a successful genomic parasite (50). In each case, TE targeting represents a balance between successful amplification of the TE and minimization of the negative consequences of that amplification. In some cases, this results in TE insertions that rarely result in deleterious mutations. In others, it is likely that the costs of those mutations are outweighed by the benefits with respect to successful amplification.

**Figure 6.**
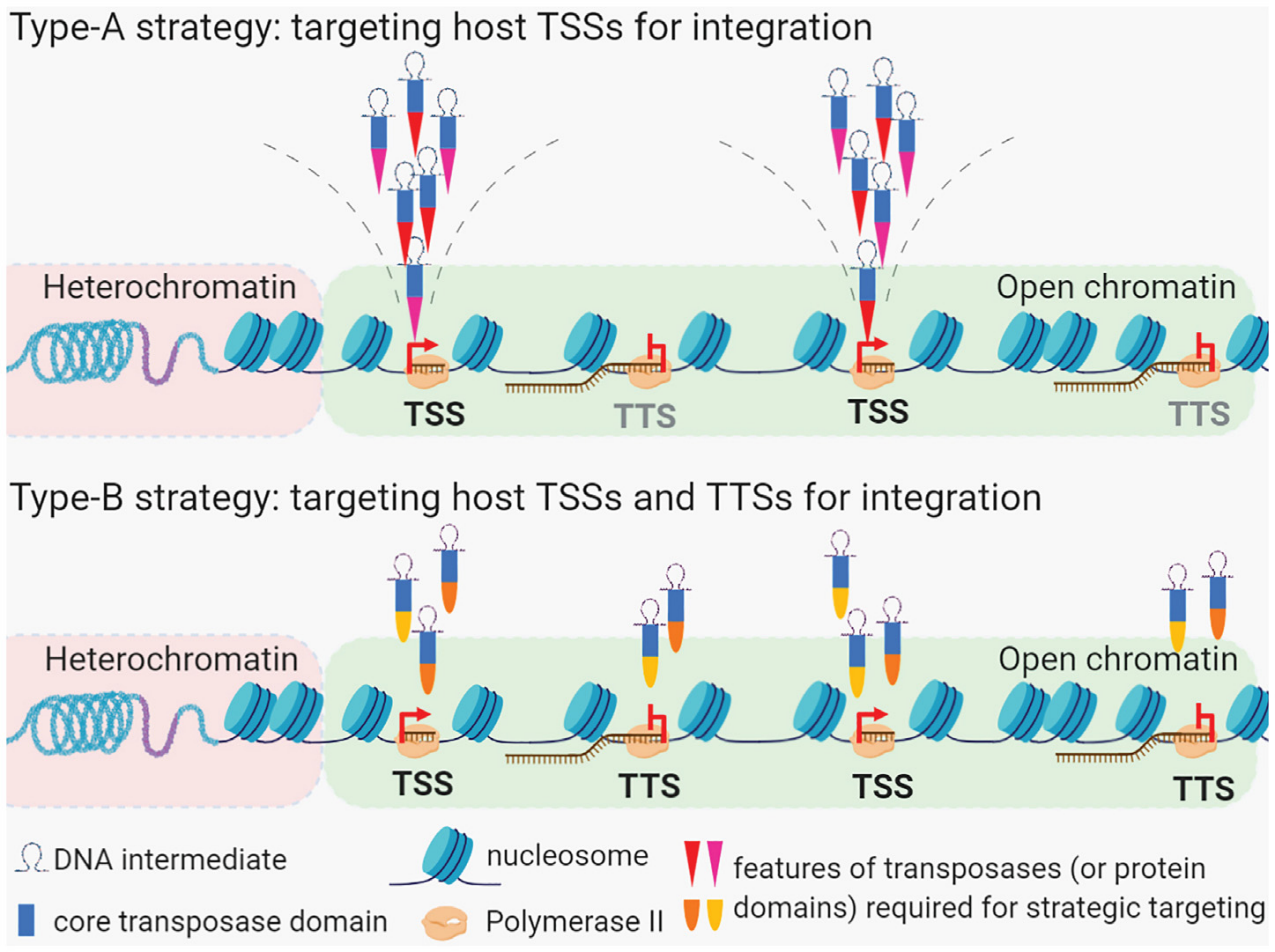
Classification of TE targeting strategies in context of host transcription. A proposed model depicting occupation of TEs at genomic locations with different expression characteristics. Type-A and type B transposons primarily target open chromatin regions where active transcription occurs. Integration of the type-A transposons associates with RNA Pol II transcription initiation. Factors that directly mediate transposase-recruitment to TSSs are yet to be identified. Targeting of the type-B transposons associates with both RNA Pol II transcription initiation and termination.

Our comparative genomic analysis of multiple *de novo* transposon collections has revealed two types (A and B) of transcription-associated TE integration strategies (Figure 6A). The type-A precision-targeting strategy, employed by two of the most active plant and animal transposons, *Mu* and *P* elements (9), is characterized by a very tight association between TE integration and Pol II-dependent transcription initiation. These TEs are strongly and specifically enriched near annotated TSSs, particularly in genes that express at a high level. Further, in maize, insertions of *Mu* elements insert preferentially into genes expressing at a high level in the actively dividing cells that are most likely to give rise to germinal lineages.

In contrast to type-A strategy, sites targeted by TE families employing the type-B strategy (*Ds* and *Pb*) are enriched at both TSSs and TTSs of medium expressed genes, and the enrichment levels of TE insertions near TSSs are lower for type-B than for type-A elements (Figure 6A, B). The type-A and type-B strategies have been named according to the single-peak and double-peak shapes of representative TE distribution curves near annotated genes reminiscent of the single- and double-hump nature of Arabian camels and Bactrian camels, respectively. Given that transposases encoded by the different TE families we have analyzed are distantly related phylogenetically (51), the transcription-associated type-A and type-B strategies suggest a process of convergent evolution among different TE families in plants and animals. Although it is formally possible that targeting of these different classes of TEs predated their divergence, we suggest that it is more likely that selection independently favored similar targeting strategies. To our knowledge, this is the first comparative study showing such relationships between TEs in both plants and animals.

Those TEs lacking an association with either Pol II transcription initiation or termination fall in the type-C group. Pol II-independent integration can be transcription-independent or transcription-associated. Reminiscent of the Sleeping Beauty transposons reported previously (52), *Tos17* insertions do not show an association with either TSSs or TTSs, but are enriched in gene bodies of genes that express at a moderate level (Figure 3C, Supplementary Figure S1C and S1F). In contrast, *Mb* insertions show a mild association with TTSs, but not TSSs (Figure 1B and E) and actually exhibit a negative correlation between targeting frequencies and gene expression levels (Figure 3B). *Spm* also show targeting enrichment near genes that are expressed at medium-to-high levels, but this TE preferentially targets intergenic regions in host genomes. Overall, the type-C strategy may involve genome targeting mechanisms that are only indirectly related to host transcriptional activities.

Previous reports provided evidence for an ‘open-chromatin’ targeting model for some DNA transposons (12,53). Our results challenge the universality of this model because it does not fully account for the type-A integration strategy, although the insertion sites of precision-targeting type-A TEs certainly do co-localize to some extent with a set of chromatic modifications associated with open chromatin (12). The majority of open chromatin regions lie just upstream of TSSs and downstream of TTSs in both plants and animals (54,55). Occupancy of Pol II at or near both TSSs and TTSs has also been reported in both maize and *Drosophila* (56,57). The distribution of *Mu* and *P* element insertions is reminiscent of Pol II occupancy near TSSs, but not near TTSs, indicating that integration of the typical type-A TEs is strongly associated with transcription initiation, and not simply with the occupancy of Pol II. Further, we demonstrated that the type-A transposition strategy is specific to Pol II, but not other RNA polymerases like Pol I, Pol III or plant-specific Pol IV and Pol V (Figure 2). *P* integration near TSSs has been attributed to enrichment of replication origins in those regions, suggesting that an association with Pol II transcription initiation is indirect (13). The proposed model involves targeting of unfired replication origins by transposons in combination with homologous repair of excision sites following replication. While this model provides an attractive mechanism for increasing element copy number, an association with replication origins does not immediately account for the observed correlations of both *P* and *Mu* targeting with levels of gene expression (Figure 3A, B). Instead, the extreme specificity of *P* and *Mu* elements suggests that they are targeted via some form of tethering. This is consistent with the observation that type-A *Tf1* retrotransposons in fission yeast are known to target the 5′ end of Pol II-transcribed genes via interaction between the integrase and the DNA binding protein Sap1, which causes replication fork arrest (26,27). Similarly, a comparative analysis of insertion site profiles has revealed that the Mouse Leukemia Virus (MLV) and the *piggyBac* transposon in human cell lines are targeted specifically to acetylated histones near TSSs via a tethering mechanism dictated by chromatin-bound bromodomain and extraterminal (BET) domain proteins that bind to acetylated H3 and H4 near TSSs (24). Future characterization of proteins or chromatin features associated with *Mu* and *P* element transposases will provide mechanistic insights into precision targeting of these elements as well.

Our analysis highlights two features that can facilitate TE survival and rapid proliferation: tissue-specific transposition and minimization of negative impacts on nearby gene function due to precision targeting. Certainly, tissue-specific transposition is true for *P* elements, which only express functional transposase in the germline (58). In plants, rapid and heritable amplification of TEs would also be facilitated by meristematic-tissue-specific transposition because actively dividing plant cells (particularly in floral tissues and meristems) are more likely to be transmitted to the next generation than those that are not. In this regard, we have observed clear targeting preferences of *Mu* and *Ds* elements for genes that express at targeted levels (highly expressed for *Mu* and expressed at a medium level for *Ds*) in meristem-enriched tissues (Figure 4B). Given that the very large number of somatic *Mu* insertions we have identified showed insertion preferences similar to germinally transmitted *Mu* insertions, *Mu* elements may be primarily avoiding insertions into genes that express at high levels primarily in terminally differentiated cells rather than targeting ‘germinal’ lineages.

The type-A (TSS-targeting) strategy employed by *Mu* and *P* elements endows these TEs with the capacity to exploit a permissive environment with respect to transcription of autonomous elements. This is particularly important for the survival of TEs in heterochromatin-rich genomes such as the maize genome. This strategy has the potential to cause deleterious effects on host gene expression and function. Indeed, we found that *Mu* insertions into CDS regions, which are most likely to be disruptive, were more frequent by 7–9-fold than random insertions. Despite of this, there was a much higher enrichment (64–90-fold) for *Mu* insertions near TSSs, and the majority of *Mu* insertions in promoters and 5′ UTRs have minimal to no effect on gene expression. This suggests that *Mu* elements are actually much less mutagenic than one might expect given their propensity to target genic regions because the reduction of host fitness is minimized due to a tight association between *Mu* element insertions and TSSs. Given this, and given that the vast majority of genic *Mu* element insertions are into the 5′UTR, we suggests that researchers who use *Mu* as a genetic resource treat these insertions with some caution, as they are unlikely to be knockouts. *P* elements also rarely insert into CDSs, likely because they are also precisely targeted to TSSs, although the effects caused by promoter and 5′ UTR targeting by *P* elements require future evaluation. Similarly, MITE TE insertions, although they tend to be into or near genes, also have a minimal effect on gene expression, although this may be in part due to their small size (59). Collectively, these data suggest that for some TEs selection has favored insertions that are into genes but that are minimally disruptive. This historical view of *Mu* and *P* elements as highly effective mutagens has likely been shaped by the fact that many of the insertion mutations caused by these elements were identified in screens for mutant phenotypes (60,61). More broadly, nearly all of the known active TEs in higher eukaryotes were first identified due to their mutagenic effects. It may well be that there are many additional active TEs in natural populations that have yet to be identified because they only rarely cause visible mutations.

Our analysis of older MULE insertions in both maize and rice suggests that 5′ UTR insertions of these elements are eventually purged from the genome, indicating that TEs that target 5′ UTRs are subject to purifying section in the long run. Interestingly, the purging appears to be much less efficient for proximal promoter MULE insertions, which are still present in high numbers in both species.

TE families occupy distinct genomic niches by employing distinct strategies for integration. This, in turn has influenced the degree to which TEs have affected host gene function and, ultimately, host genome evolution. TEs have also proved to be invaluable tools, both as mutagens and as transformation vectors (62). A deeper understanding of the ways in which TEs target particular regions of the genome for integration promise to make those tools both more effective and more precise.

## DATA AVAILABILITY

The RNA-seq data of B73/Mo17 hybrids and Museq data generated in this study have been deposited in the Gene Expression Omnibus (GEO) data bank, accession codes PRJNA556108 and GSE146647.

## Supplementary Material

gkaa370_Supplemental_Files
