# Supplementary material for: Transposable elements employ distinct integration strategies with respect to transcriptional landscapes in eukaryotic genomes
Source: Nucleic Acids Res. 2020 May 22;48(12):6685–98. doi: 10.1093/nar/gkaa370 (PMC7337890; doi:10.1093/nar/gkaa370)
Supplement: gkaa370_Supplemental_Files [file gkaa370_supplemental_files.zip › Supplemental_Figures.pdf]

Table S1. Hot-spot genes frequently targeted by Mu elements.

Table S2. Coordinates of randomly selected loci and de novo insertions in maize (AGPv4 B73).

Table S3. Coordinates of randomly selected loci and de novo insertions in Drosophila (FB2014_03, R5.57).

Table S4. Coordinates of randomly selected loci and de novo insertions in rice (Oryza_sativa.IRGSP-1.0.42).

Table S5. Coordinates of ancient (natural) transposon insertions in rice, maize and Drosophila in this study.

Table S6. rRNA and tRNA gene sets in maize and Drosophila analyzed in this study.

Table S7. FPKM values for RNA-seq datasets collected in maize, Drosophila and rice for expression analysis in this study.

Table S8. Oligo sequences used for amplicon PCR for transposon profiling and Miseq-based sequencing of RT-PCR products.

Figure S1

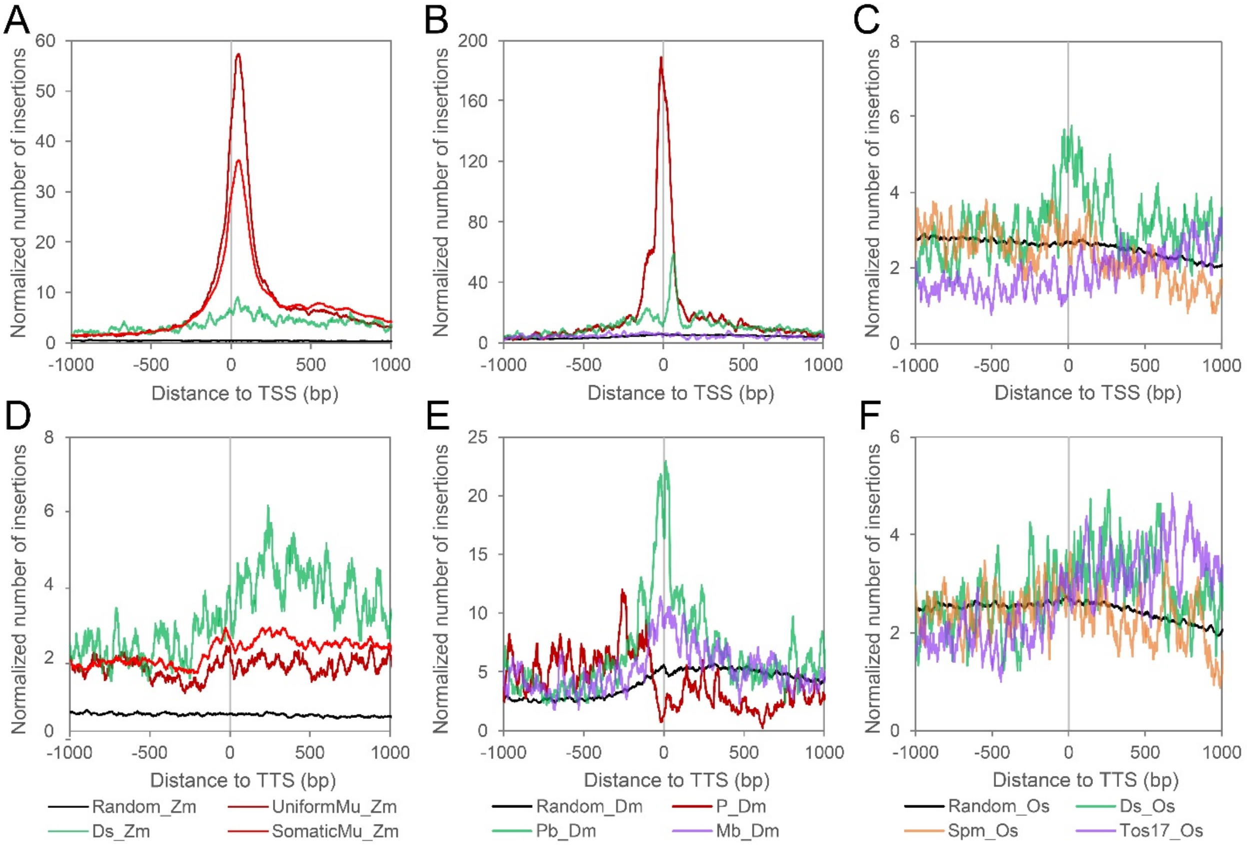

Figure S1. Characterization of distribution of *de novo* transposon insertions that are near genes.

The figure shows normalized numbers of insertions around TSSs and TTSs of annotated genes within 1 kb upstream and downstream of TSS and TTS. The insertion numbers at each distance was smoothed by computing the mean in a 30-bp rolling window. For both TSS and TTS plots, normalized numbers of genic insertions were plotted on the positive X-axis coordinates and normalized numbers of intergenic insertions were plotted on the negative coordinates. Thus, positive values for both plots represent insertions into the gene body. Randomly selected loci were used as a background control to take into account the fact that although insertions were plotted up to one kilobase pairs (kb) away from the TSSs and TTSs. Panels in the upper and bottom rows show metaprofiles of transposon insertions in maize (A, D), Drosophila (B, E) and rice (C, F) for regions surrounding the gene TSS and TTS, respectively.

Figure S2

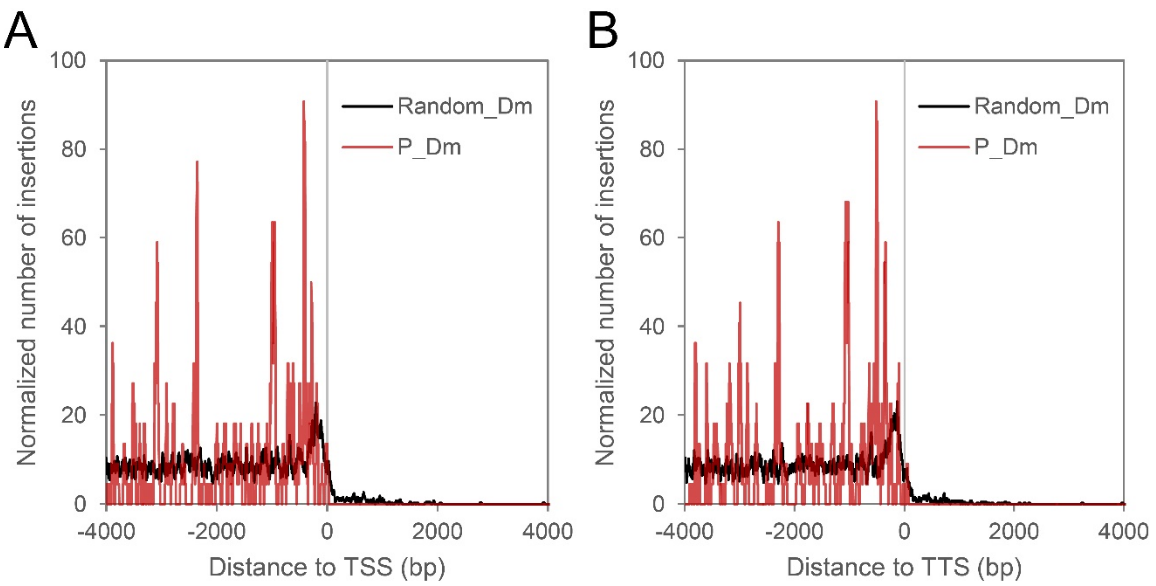

Figure S2. Distribution of *de novo* P element insertions surrounding TSS (A) and TTS (B) of tRNA and rRNA genes.

The figure shows normalized numbers of insertions around TSSs and TTSs of annotated tRNA and rRNA genes. The insertion numbers at each distance were smoothed by computing the means in 30-bp rolling windows. Normalized numbers of genic insertions were plotted on the positive X-axis coordinates and those of intergenic insertions were plotted on the negative coordinates.

Figure S3

A

Gene Set Name(NO. Genes)	Description	Category	NO. Genes in Overlap (k)	p value	FDR
PROTEIN_BINDING(2087)	GO:0005515 protein binding, GOslim:molecular_function	GO_MF	345		2.96E-13
PROTEIN_KINASE_ACTIVITY(1459)	GO:0004672 protein kinase activity, GOslim:molecular_function	GO_MF	264	1.08E-16	2.96E-13
PROTEIN_SERINE/THREONINE_KINASE_ACTIVITY(1532)	GO:0004674 protein serine/threonine kinase activity, GOslim:molecular_function	GO_MF	272	2.83E-16	2.96E-13
PROTEIN_PHOSPHORYLATION(1626)	GO:0006468 protein phosphorylation, GOslim:biological_process	GO_BP	275	3.21E-14	6.23E-11
SIGNAL_TRANSDUCTION(240)	GO:0007165 signal transduction, GOslim:biological_process	GO_BP	66	5.80E-11	5.63E-08
RESPONSE_TO_HORMONE_STIMULUS(40)	GO:0009725 response to hormone stimulus, GOslim:biological_process	GO_BP	19	7.80E-07	5.05E-04
TRANSFERASE_ACTIVITY,_TRANSFERRING_GLYCOSYL_GROUPS(95)	GO:0016757 transferase activity, transferring glycosyl groups, GOslim:molecular_function	GO_MF	28	5.98E-06	4.69E-03

B

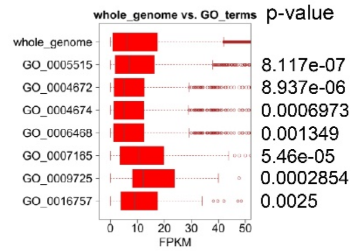

C

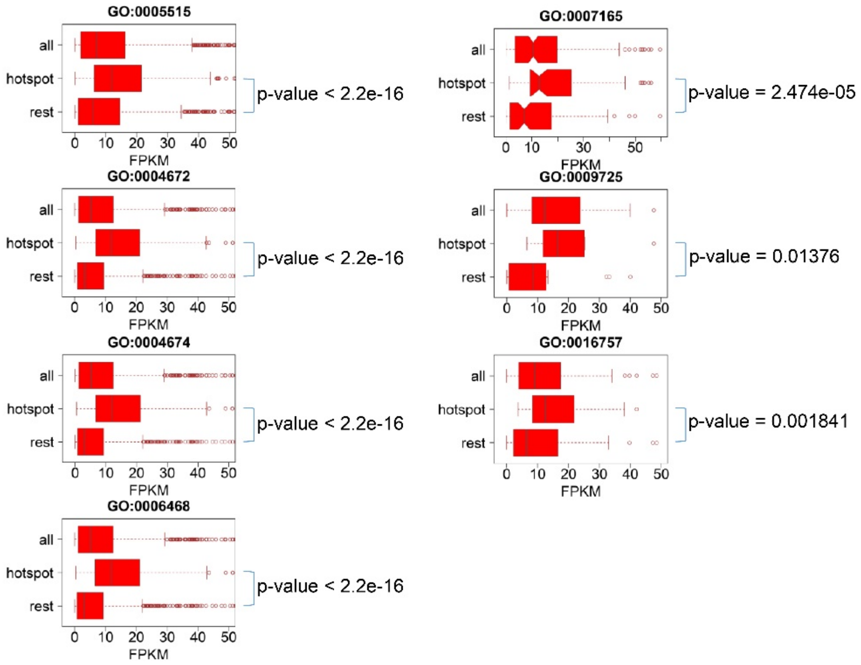

Figure S3. GO analysis on the hotspot genes frequently targeted by *Mu* elements

A. A list of top enriched categories of the hotspot gene set as ranked by the webserver agriGO.

B. Distribution of gene expression values. Box plot shows distribution of transcript values of the seven GO gene sets and the whole genome genes. Kolmogorov-Smirnov tests were performed comparing each GO gene set and the whole-genome gene set. P values are indicated next to the box plots.

C. Box plots show distribution of transcript values of the overall genes, the hotspot and non-hotspot genes in each category. Kolmogorov-Smirnov tests were performed comparing the hotspot gene set and the rest genes in each category. P values are indicated next to the box plots.

Figure S4

A

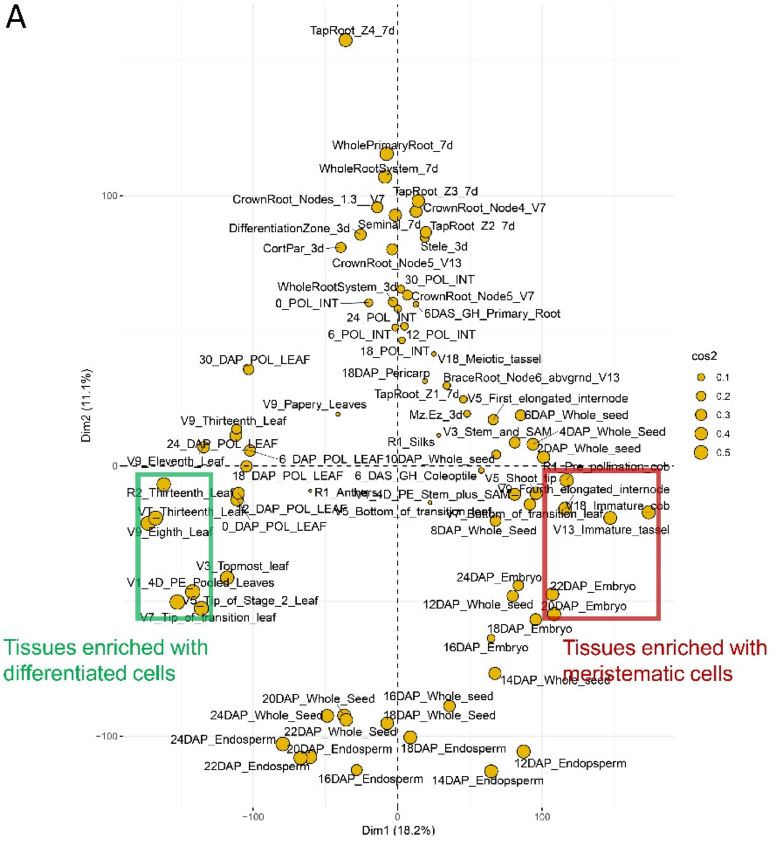

B

Type	Tissue selected
meristematic	20DAP_Embryo
meristematic	22DAP_Embryo
meristematic	R1_Pre_pollination_cob
meristematic	V13_Immature_tassel
meristematic	V18_Immature_cob
meristematic	V9_Fourth_elongated_internode
differentiated	R2_Thirteenth_Leaf
differentiated	V1_4D_PE_Pooled_Leaves
differentiated	V5_Tip_of_Stage_2_Leaf
differentiated	V7_Tip_of_transition_leaf
differentiated	V9_Eighth_Leaf
differentiated	VT_Thirteenth_Leaf

Figure S4. *Mu* insertion profiles differ in meristematic and differentiated tissues

A. Principal component analysis (PCA) of the maize tissue-specific gene expression. Red frame marks tissues enriched with meristematic cells, and green frame marks those enriched with differentiated cells.

B. A list of 6 meristematic tissues and 6 differentiated tissues.

Figure S5

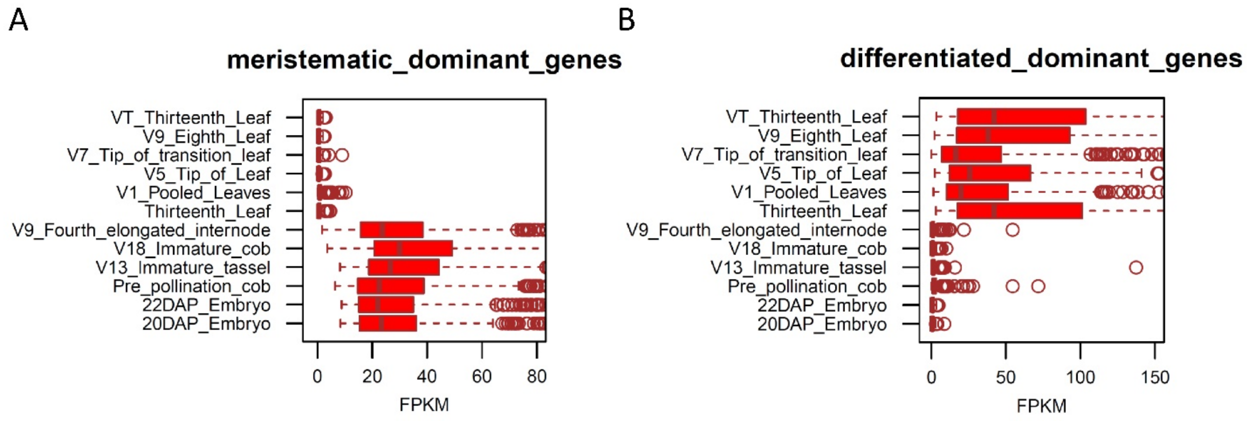

Figure S5. Box-plot diagrams showing differential expression of a set of meristematic-tissue-dominant genes and a set of differentiated-tissue-dominant genes in selected meristematic tissues (6 on the bottom) and differentiated tissues (6 on the top).

Figure S6

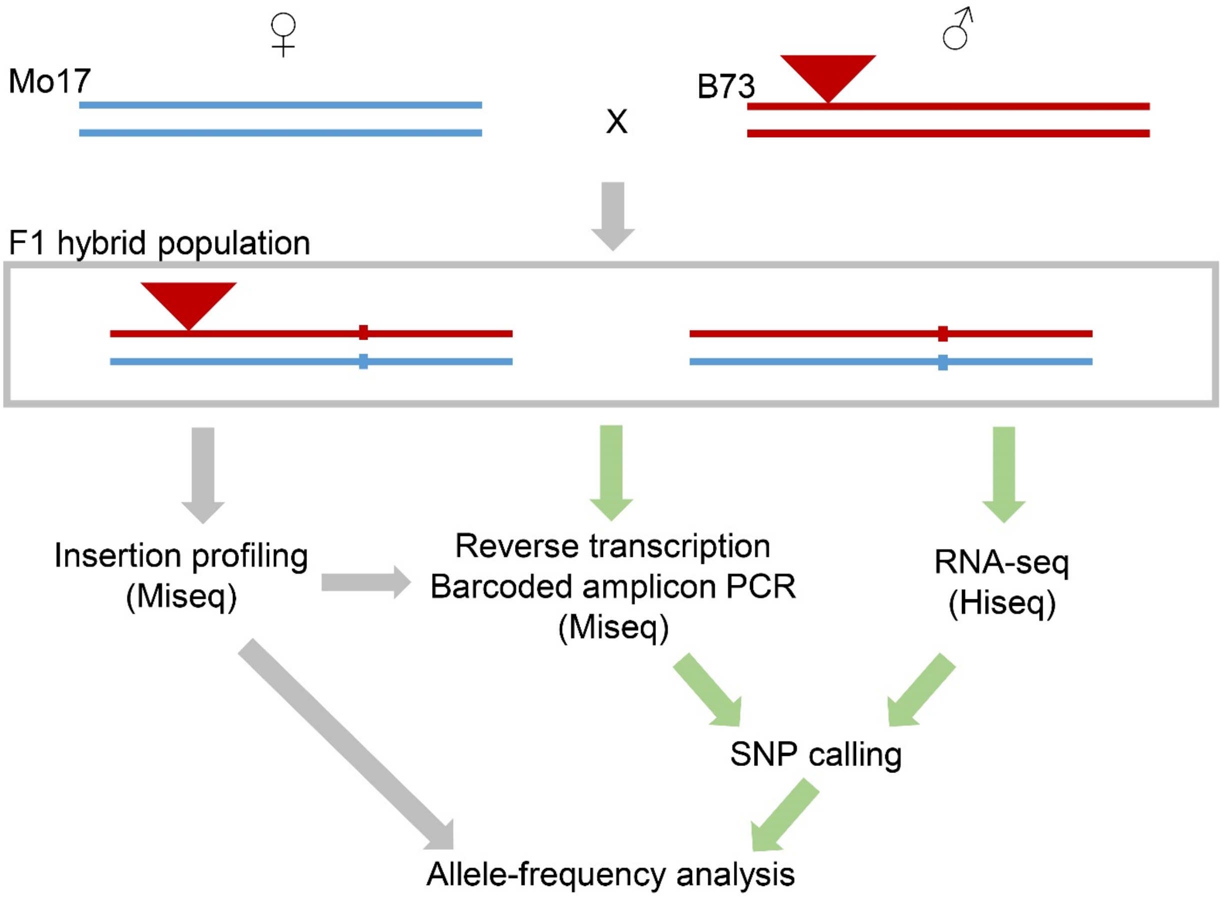

Figure S6. A scheme of the genetic crosses made to generate for hybrid Mu-segregating population and quantification of allele frequency based on high-throughput sequencing.
